# Supplementary material for: Complexome profiling of the Chlamydomonas psb28 mutant reveals TEF5 as an early PSII assembly factor
Source: Plant Cell. 2025 Mar 17;37(6):koaf055. doi: 10.1093/plcell/koaf055 (PMC12164590; doi:10.1093/plcell/koaf055)
Supplement: koaf055_Supplementary_Data [file koaf055_supplementary_data.zip › psb28_interactive.zip!/plots/intensity/qualityPlots/peptides/HSP90C_Cre12.g514850.t1.2.html]

### Cre12.g514850.t1.2

Trivial name: HSP90C  
SeqCoverage [median]: 30.10  
Mass: 89.35 kDa  
  
 

| Sequence | MissedCleavage | Mass [Da] | wtIntensity | muIntensity | Score |
| --- | --- | --- | --- | --- | --- |
| AAEAAAVPK | 0 | 826.45 | 1614.03 | 6043.48 | 122.74 |
| AAPFMEAMVAK | 0 | 1164.57 | 0.00 | 827.73 | 47.60 |
| AEDSLTSLDEYVGR | 0 | 1553.72 | 720.06 | 0.00 | 160.36 |
| AGSHQYSIREDEAKDLVR | 2 | 2073.02 | 0.00 | 1731.52 | 59.90 |
| AMEYMK | 0 | 771.33 | 0.00 | 565.67 | 79.45 |
| EILQESR | 0 | 873.46 | 1988.74 | 7838.88 | 120.65 |
| ELISNASDALDKAR | 1 | 1501.77 | 0.00 | 3822.99 | 125.61 |
| EQLLSNLGTIAR | 0 | 1313.73 | 2000.12 | 0.00 | 112.13 |
| FLSLTDPSVLAGREELDIR | 1 | 2130.13 | 1540.59 | 0.00 | 41.09 |
| FSSSKAEDSLTSLDEYVGR | 1 | 2089.98 | 502.36 | 0.00 | 59.51 |
| GTLVIEDSGIGMSR | 0 | 1433.72 | 1469.85 | 4667.40 | 155.97 |
| GVVDSSDLPLNVSR | 0 | 1456.75 | 5095.95 | 12809.13 | 165.66 |
| KVVDEEATK | 1 | 1017.53 | 960.38 | 2589.25 | 98.05 |
| SIEMLEELAGK | 0 | 1218.62 | 0.00 | 1028.47 | 75.38 |
| SQALGDAR | 0 | 816.41 | 648.17 | 4580.86 | 118.50 |
| TIYYMAADSVAAAR | 0 | 1501.72 | 352.75 | 1013.57 | 90.69 |
| YGPDKNGPQYELVDVSK | 1 | 1907.93 | 0.00 | 547.70 | 49.46 |
| YGVIEDTENRER | 1 | 1479.70 | 3766.76 | 7279.05 | 84.66 |
|  |  |  | 20659.8 | 55345.7 |  |
